# Association of Food Desert Residency and Preterm Birth in the United States

**DOI:** 10.3390/ijerph21040412

**Published:** 2024-03-28

**Authors:** Chanell O. Haley, Chelsea R. Singleton, Lily E. King, Lauren Dyer, Katherine P. Theall, Maeve Wallace

**Affiliations:** 1 Department of Behavioral, Social and Population Sciences, Tulane University School of Public Health and Tropical Medicine, New Orleans, LA 70112, USA; csingle1@tulane.edu (C.R.S.); lking9@tulane.edu (L.E.K.); lauren.dyer2@la.gov (L.D.); ktheall@tulane.edu (K.P.T.); mwallace@tulane.edu (M.W.); 2 Mary Amelia Center for Women’s Health Equity Research, Tulane University School of Public Health and Tropical Medicine, New Orleans, LA 70112, USA; 3 Department of Epidemiology, Tulane University School of Public Health and Tropical Medicine, New Orleans, LA 70112, USA

**Keywords:** food desert, food insecurity, preterm birth, inequities, Black/African American

## Abstract

Introduction: Food deserts are a major public health concern. Inadequate access to healthy food has been associated with poor nutrition and the development of dietary related chronic conditions. Objective: To determine the association between geographic access to nutritious food and preterm birth and whether gestational hypertension mediates this relationship. Methods: Food access data was retrieved from the U.S. Department of Agriculture Food Access Research Atlas (2019) and used to quantify the percentage of Census tracts within each county that were food deserts: low-income tracts with limited access to grocery stores, supermarkets, or other sources of healthy, nutritious foods. These data were merged with US birth records from 2018 to 2019 by using the maternal county of residence (*n* = 7,533,319). We fit crude and adjusted logistic regression models with generalized estimating equations to determine the association between living in a food desert and the odds of preterm birth. We conducted a secondary within-group analysis by stratifying the fully adjusted model by race for non-Hispanic White and non-Hispanic Black birthing people. Results: In the fully adjusted model, we found a dose–response relationship. As the prevalence of tract-level food deserts within counties increased, so did the likelihood of preterm birth (mid-range: odds ratio (OR) = 1.04, 95% confidence interval (C.I.) 1.01–1.07; high: OR = 1.07, 95% C.I. 1.03–1.11). Similar results were seen in the White–Black stratified models. However, a disparity remained as Black birthing people had the highest odds for preterm birth. Lastly, gestational hypertension appears to mediate the relationship between nutritious food access and preterm birth (natural indirect effect (NIE) = 1.01, 95% CI = 1.00, 1.01). Conclusion: It is salient, particularly for Black birthing people who experience high rates of adverse birth outcomes, that the role of food desert residency be explored within maternal and child health disparities.

## 1. Introduction

Despite recent national and local efforts to expand access to healthy food in the U.S., several geographic areas continue to have limited access [1,2]. Areas with limited access to retailers that offer affordable nutritious foods (e.g., supermarkets and grocery stores) are considered food deserts. Residing in a food desert has been linked to food insecurity, which occurs “when people lack secure access to sufficient amounts of safe and nutritious food for normal growth and development and an active and healthy life” [3]. Food deserts have been found to spatially co-exist with food swamps, areas inundated by retailers that primarily offer inexpensive, calorically dense, and less nutritious foods (e.g., corner stores, fast food restaurants) [4,5]. Similar to food deserts, food swamps are associated with adverse health risks such as obesity [6]. Limited healthy food access, coupled with excessive access to unhealthy foods, may promote poor dietary behaviors [7] and increase the likelihood of diet-related chronic diseases such as hypertension and diabetes [8,9].

National and local data have consistently shown that a lack of healthy food access disproportionately affects low-income and racialized communities. Census tract data indicate low-income Black communities often have few supermarkets but several corner stores and liquor stores [10]. Thus, they are more likely to be labeled a food desert or food swamp [10,11]. The healthy food access disparities impacting racialized communities may be more distally caused and exacerbated by systemic determinants such as racial segregation, chronic disinvestment, and poor community infrastructure, all of which have great potential to affect individuals and communities beyond health behaviors [12]. 

Poor, healthy food access can negatively affect maternal health outcomes within socially and economically disadvantaged populations, ultimately leading to health inequities. Inadequate food access during pregnancy increases the likelihood of developing at least one morbid condition [9]. One of the leading causes of maternal morbidity and mortality is hypertension disorders of pregnancy (HDP) [13], which occurs in 5% to 10% of all pregnancies [14]. Health organizations have categorized HDP into four primary types: gestational hypertension, chronic hypertension, chronic hypertension with superimposed preeclampsia, and preeclampsia/eclampsia [15]. Studies suggest that HDP, such as gestational hypertension (gHTN), may be significant factors in the occurrence of preterm birth [16]. Arfandi et al. (2023) reported that although preterm birth risk may differ by the type of HDP, each form of HDP carries a significant risk for preterm birth [17]. 

In the U.S., the rates of preterm birth rose 12% between 2014 and 2022 [18]. Globally, preterm birth is the leading cause of death for children under the age of five [19,20]. It may also affect physical/neurological development and increase the risk of developing health conditions [21]. Preterm birth remains a public health concern, and racial inequities in preterm birth have not abated, with preterm birth rates 50% higher among non-Hispanic Black infants than non-Hispanic White infants [22]. 

The connection between geographic access to healthy food retailers and chronic conditions (i.e., obesity, hypertension, and diabetes) is well established [23,24]. Although proper diet and nutrition have been recognized in maternal and child health, proximity to healthy food has been an understudied determinant for maternal and infant health outcomes but may impact diet during the perinatal and postpartum periods as well as long before pregnancy. Racism has shaped U.S. neighborhoods [25] and continues to shape both built and social neighborhood conditions, including food access and insecurity [26]. Given the pervasive racial inequities in not only maternal and infant health outcomes in the U.S. but also neighborhood exposures, it is paramount that we better understand the role of these more distal neighborhood conditions in shaping these outcomes and the pathways through which that may occur. 

Using a rigorously adjusted model as well as within-group and mediation analyses, this study aims to examine the association between residing in a U.S. Department of Agriculture (USDA)-designated food desert and preterm birth and the mediating effect of gHTN in this association. We hypothesized that pregnant persons residing in food deserts have a higher risk of preterm birth and that this association would be explained, in part, by gHTN.

## 2. Materials and Methods

### 2.1. Study Population

An analysis was conducted using cross-sectional data including all live births in the US for the years 2018–2019, data obtained from the National Center for Health Statistics. Birth records with missing geographical identifiers for maternal county of residence or births that occurred in U.S. territories (Guam, Virgin Islands, Puerto Rico, American Samoa, and Northern Marianas) were excluded from the analyses (*n* = 25,797), resulting in an analytic sample size of *n* = 7,533,319. As all data were deidentified, this study was deemed exempt by the Tulane University Institutional Review Board.

### 2.2. Exposure Variable

Food desert data were retrieved from the USDA Food Access Research Atlas (2019), which provides a spatial overview of low-income and low-access (LILA) census tract-level food access data. Included in these data is a tract-level indicator designating both LILA areas: low-income tracts where the populace of at least 500 people and/or at least one-third of the census tract live beyond a specified distance to a grocery store (>1 mile for urban communities; >10 miles for rural communities) [27]. A census tract is designated as low-income if it has a poverty rate of at least 20% or a median family income at or below 80 percent of the metropolitan area or state median income level [27]. Additional LILA tract-level indicators of food access were also examined: half mile (urban) and 10 miles (rural), 1 mile (urban) and 20 miles (rural). We counted the number and percentage for each LILA tract-indicator within each US county and operationalized county-level food desert variables to identify counties by the prevalence of LILA tracts within them (low: 0–33.4%; mid-range: 33.5–66.6%; and high: 66.7–100.0%). The food access indicator variables were linked to birth records by a geographic identifier for the maternal county of residence (Federal Information Processing System codes). 

### 2.3. Outcome

Using the information provided on the birth record, preterm birth (occurring <37 weeks gestation) was measured as a binary outcome (yes or no), denoting whether an infant was delivered prior to 37 weeks gestation. 

### 2.4. Individual-Level Covariates

Sociodemographic variables associated with pregnancy and birthing outcomes were retrieved from birth records and included in the fully adjusted model: maternal age (≤19, 20–34, and ≥35), education attainment (less than high school, diploma or equivalent, some college, or college degree), month prenatal care began (no prenatal care, 1st trimester, 2nd trimester, or 3rd trimester), and previous preterm birth (yes or no).

### 2.5. County-Level Covariates

We controlled for additional geographic and socioeconomic characteristics that differ by county, which may be associated with accessibility to health care services and maternal health outcomes. Data were obtained from the American Community Survey’s 2019 (5-year estimates) and included the percentage of families living below the poverty level, and the median household income. Additionally, we controlled for whether a county/parish was rural or urban based on the 2010 Census Urban Rural Classification Scheme.

### 2.6. Mediator

Gestational hypertension was defined as a blood pressure of ≥140/90 mmHg without or with proteinuria of no greater than trace levels after 20 weeks of gestation [28]. A diagnosis of gestational hypertension, or pregnancy-induced hypertension, was provided by birth record data. A binary response (yes or no) was used to indicate whether a pregnant person had the condition. We proposed a mediation pathway where gestational hypertension partially explains the relationship between food desert residency and preterm birth. 

### 2.7. Statistical Analysis

Frequency analyses were used to describe the characteristics of the study population. Statistical analyses were performed using SAS version 9.4 (SAS Institute, Cary, NC, USA). To estimate the association between living in a food desert and preterm birth, we fit crude and adjusted modified logistic regression models with generalized estimating equations, using the PROC Genmod function, and cluster-robust standard errors to account for multiple births occurring within the same county to obtain odds ratios (OR) and 95% confidence intervals (C.I.). In addition to the fully adjusted model, we conducted within-group analyses to provide insight on contributing social determinants and behaviors that may be lost in comparative analyses between racial groups [29]. This was completed by fitting the fully adjusted model for births to White and Black people separately.

A mediation analysis was examined using SAS macro statistical software that accounts for confounding between exposure, mediator, and outcome as well as interaction [30]. We were particularly interested in the natural indirect effect (NIE), or the effect of changing the mediator value given exposure is fixed, and the natural direct effect (NDE), or the effect of food desert exposure on PTB given the mediator, gHTN, takes the level it would have been under the absence of exposure. Spatial mapping and tests for global spatial clustering with Moran’s I were performed with GIS in ArcPro (v3.0.3, ESRI, Inc., Redlands, CA, USA). In general, the global Moran’s I ranges from −1.0 to 1.0, with positive values indicating the occurrence of similar values of the variable over space (either high or low values) and negative values indicating that there are areas with dissimilar values and no spatial clustering (often referred to as a checkerboard pattern). 

## 3. Results

A large percentage of the study population (76.8%) were between the ages of 20 and 34, and 41.6% held college degrees at the time of birth. Approximately half (51.8%) identified as non-Hispanic White, with 14.7% racially identifying as non-Hispanic Black. Most (96.4%) had no previous preterm births, and 87.6% resided in urban counties. A total of 7.5% reported a diagnosis of gHTN; of those with gHTN, 55.4% identified as non-Hispanic White and 17.7% identified as non-Hispanic Black. The mean percentage of county tracts designated as low income/low access food desert indicators of 1 mile (urban) and 10 miles (rural) from a supermarket was 13.1% (Table 1).

**Figure 1:**
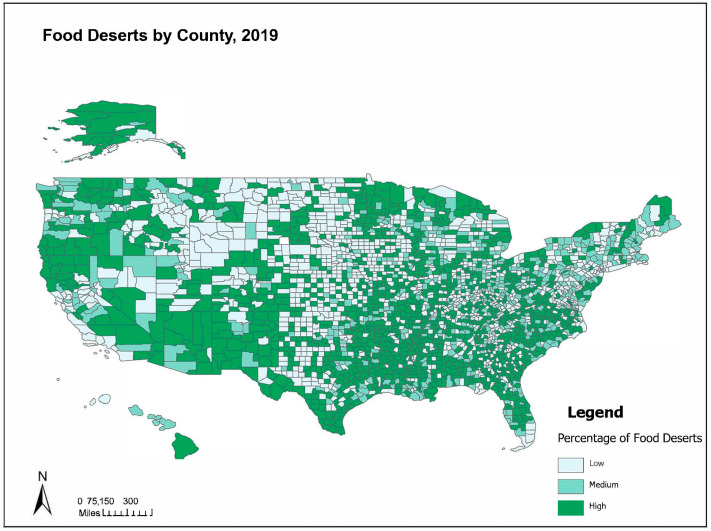
U.S.-designated food deserts by county.

The prevalence of high food deserts in the U.S. across counties is substantial, as shown in Figure 1, with significant clustering (Moran’s I = 0.178; *p* < 0.0001) across the U.S. and a high proportion of deserts in the Gulf South and Mississippi Delta, southern border, and southwest region.

In the fully adjusted model, among persons residing in communities within 1 mile (urban) and 10 miles (rural) of the nearest grocery store, there was a slight increase in the likelihood of preterm birth for counties with a mid-range (OR = 1.04; 95% C.I. 1.03–1.05) and high (OR = 1.07; 95% C.I. 1.06–1.08) percentage of tract-level food deserts (Table 2). Similar results were seen in the fully adjusted stratified model. The food desert indicator, LILA 1 mile (urban) and 10 miles (rural), showed an increased likelihood of preterm delivery for non-Hispanic Black births as the percentage of census-tract level food deserts increased within a county; mid-range (OR = 1.06; 95% C.I. 1.04–1.08) and high percentage (OR 1.10; 95% C.I. 1.08–1.12). However, for non-Hispanic Whites, the mid-range percentage was not statistically significant. The high percentage was associated with a significant likelihood of preterm birth (OR = 1.06; 95% C.I. 1.05–1.07) (Table 3). 

**Figure 2:**
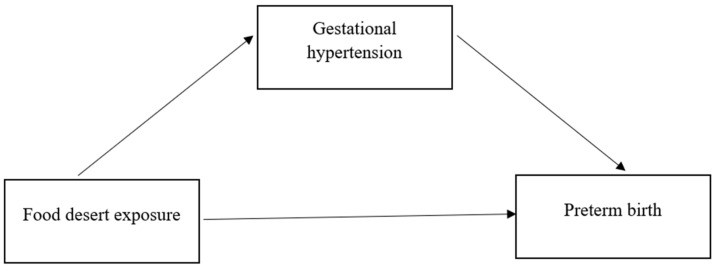
Conceptual design of the mediation analysis pathway.

Prior to the mediation analysis, regression analyses were completed to determine the association between the variables (Figure 2). The level of county food desert residency for tract-levels with mid-range (OR = 1.13; 95% C.I. 1.02–1.24) and high (OR = 1.16; 95% C.I. 1.05–1.27) percentages had a significant association with gHTN. Similarly, women with gHTN were 3.0 times more likely to experience preterm birth (OR = 3.00; 95% C.I. 2.90–3.05). We also observed a significant indirect mediating effect of gHTN between food desert residency and preterm birth, both in crude (NIE = 1.02; 95% CI = 1.01, 1.02) and adjusted (NIE = 1.01; 95% CI = 1.00, 1.01) models, with approximately 5% of the total effect of food deserts on preterm birth eliminated by prevention of gHTN. The natural direct effect in the adjusted model was also significant (NDE = 1.08; 95% CI = 1.07, 1.09), indicating that there would be an 8% increase in PTB for those in higher food desert tracts in the absence of gHTN. 

## 4. Discussion

Our findings add to the evidence of social and structural factors that may adversely impact reproductive health and birthing outcomes. Within our fully adjusted analysis, we determined that residing in a USDA-designated food desert increased the likelihood of preterm birth in a dose–response manner. Our results were consistent with existing studies that linked inadequate access to healthy foods to poor maternal health and birth outcomes [9,31,32]. Furthermore, residing in a food desert has been associated with health implications beyond conception health and birthing outcomes. Food desert residents were found to have decreased breastfeeding initiation rates compared to individuals who did not reside in USDA-designated food deserts [33]. 

In the racially stratified models, most of the food desert indicators were found to increase the likelihood of undergoing a preterm birth within both racial groups, which indicates that proximity to affordable food access is impactful on the health of all birthing persons regardless of their racial identity. Although food desert exposure was significantly associated with preterm birth within each racial group, in the adjusted model, the likelihood for preterm birth for non-Hispanic Black mothers was greater than that of their racial counterparts. This indicates that in addition to social and structural factors (medical bias, medical care accessibility, psychological stress, neighborhood infrastructure, and racial discrimination) found to contribute to the high incident rate of preterm labor among non-Hispanic Black birthing persons [34,35], the food environment should further be examined as a contributing factor for racial birthing inequities.

Moreover, there was an overrepresentation of non-Hispanic Black births in food desert areas (*M* =15.33; *SD* = 12.91); the mean for non-Hispanic White mothers residing in a food desert (*M* = 13.09; *SD* = 12.10) was the same as the national average. Researchers postulate that inequitable food access stems from systemic and structural racism [26,36]. Structural racism and biased ideologies impact infrastructure, policies, and access to resources for underserved populations. Specific to the food environment, the literature shows a clear racial disparity in food access, even when controlling for socioeconomic conditions [37]. Furthermore, the occurrence of the COVID-19 pandemic may have exacerbated the apparent racial inequities in food access [38]. Dubowitz and colleagues (2021) reported a spike in food insecurity among a cohort of low-income African American food desert residents during the early phases of the pandemic [39]. Crises, such as the pandemic, shed light on the vulnerabilities within the food system and underscore the importance of equitable food access. The high prevalence of preterm birth along with the overrepresentation of Black food desert residents underscores the role that structural racism plays in our social/living environment, which, in turn, affects our health and well-being, engendering inequities at the population level. 

Similar to other studies, we found that food desert residency was associated with an increased risk of developing gestational hypertension. However, when additional covariates were added to the model, the association was no longer significant. When examining gHTN and preterm birth, the association remained significant in both the crude and adjusted models. This aligns with the existing literature, which has established gHTN as a potential risk factor for preterm birth [16,17,40]. It should also be noted that although there is a stronger statistical association between gHTN and preterm birth compared to food desert exposure and preterm birth, this does not minimize the primary findings of our study and the negative impact inadequate food access has on birthing outcomes. Furthermore, addressing distal factors (e.g., food deserts) may have a smaller association than proximal factors, but the impact on population health is greater. Distal factors often have effect sizes that are smaller in magnitude, but this study sheds light on the potential pathways between food deserts and preterm birth. 

We also observed an indirect effect between food desert exposure and preterm birth, with a large proportion of the food desert and preterm birth association mediated by gHTN. The reported prevalence of hypertensive disorders in pregnancy in the United States is 16% [41]. Based on the current study data, the prevalence of gHTN is 7.5%, which suggests underreporting, which may be reflected in our findings. However, if the prevalence of gHTN is higher within our study population than actually reported, it might also mean that the mediation effect is an underestimation as well. Nevertheless, our analysis shows a mediated effect, and while food desert areas may also be a proxy for other meso-level conditions, we did control for the level of poverty in the area. Additional pathways between food deserts and preterm birth and other reproductive outcomes should be examined. 

Inadequate food access poses a great concern for racially minoritized birthing people and those of lower income status. Health care professionals should include available survey instruments in prenatal screenings to identify food insecurity as a potential risk factor for morbid conditions and adverse birthing outcomes. Developing social support networks and discussing proper nutrition are steps health care practitioners can take to share the importance of prenatal health and weight management. Additionally, for those with pre-existing conditions, discussing ways to properly manage health conditions during pregnancy will minimize the risk of adverse health outcomes such as mortality. 

However, solutions must go beyond prenatal appointments. Residents of food deserts require adequate care and resources to improve their health. Programs and initiatives have been implemented with the hope of improving access to healthy foods in underserved communities. A community food bank implemented a mobile market pilot program to sell fresh produce and other food items in low-resourced neighborhoods [42]. An evaluation of the pilot program found an increase in vegetable intake in a few of the selected neighborhoods and, on a larger scale, has the potential to reduce nutritional inequities [42]. Crowe et al. (2018) suggest improving neighborhood conditions and making smaller grocery stores and markets more affordable for food desert residents [43]. However, interventions to improve healthy food retail in underserved communities have resulted in mixed findings [44]. Dubowitz et al. (2015) found that the development of a supermarket in a designated food desert did not improve dietary intake but positively altered residents’ perception and satisfaction of healthy food accessibility within their neighborhood [45]. As food deserts point to greater structural and systemic inequities, strategies should focus on mitigating the effects of poverty, residential segregation, and their impact on disadvantaged communities. For instance, increased minimum wages, pay equity, safe, affordable housing, and investing in neighborhood infrastructure are a few examples of anti-poverty strategies to address structural and systemic issues. 

### Limitations

Despite the strengths of this national analysis, there are several limitations. First, it is cross-sectional, which limits the temporal interpretation of our findings. Second, although we controlled for a robust set of multi-level confounders, there are factors not included in the study that have been associated with preterm birth (i.e., structural racism, stress, physical occupation, physician–patient communications, and intimate partner violence). Moreover, chronic conditions appear to be underreported in birth records; therefore, we cannot determine the full extent to which morbid conditions contributed to birth outcomes. There is also no way to determine whether people moved during their pregnancy or if relocation impacted their proximity to food access. 

## 5. Conclusions

Preterm birth is a health concern with various risk factors and mechanisms. Our findings examine inadequate access to healthy foods as a possible contributing factor to the national preterm birth rates. As healthy nutrition and dietary choices are pivotal for reproductive health, it is imperative to examine the role food access plays in birthing outcomes. Residing in a food desert raises the risk of developing or exacerbating morbid conditions, which can be a serious health concern for those of reproductive age. Although our study focuses on food accessibility and availability, researchers and practitioners must continue to address known risk factors such as inadequate medical/prenatal care, physically demanding jobs, and the community/social environment, which play a role in the overall preterm birth rates as well as the high incident rate of preterm birth among Black birthing individuals. 

## Figures and Tables

**Table 1:** Demographic characteristics.

Descriptive Statistics (*n* = 7,533,086)		
		*n* (%)
Gestational age at birth		
	>37 weeks	6,771,501 (89.8)
	<37 weeks	762,838 (10.1)
	Missing	4913 (0.1)
Previous preterm birth		
	Yes	270,492 (3.6)
	No	7,262,594 (10.1)
	Missing	4913 (0.1)
Maternal race/ethnicity		
	Non-Hispanic White	3,872,325 (51.4)
	Non-Hispanic Black	1,100,104 (14.6)
	Hispanic	1,172,677 (23.5)
	Multiracial/other	724,518 (9.6)
	Missing	69,628 (0.9)
Education attainment		
	Less than high school	928,864 (12.3)
	High School	1,932,708 (25.6)
	High school diploma/equivalent	1,479,970 (19.6)
	College educated	3,094,596 (41.0)
	Missing	103,114 (1.4)
Maternal age		
	19 and under	355,068 (4.7)
	20–34	5,788,083 (76.8)
	35 and up	1,396,101 (18.5)
Prenatal care initiated		
	No prenatal care	131,340 (1.7)
	1st trimester	5,706,639 (75.7)
	2nd trimester	1,189,864 (15.8)
	3rd trimester	331,259 (4.4)
	Missing	180,150 (2.4)
Urban residence		
	Yes	6,603,752 (87.6)
	No	934,025 (12.4)
	Missing	1475 (0.0)
Mean (SD)		
Food desert indicator 1 mile (urban) and 10 miles (rural)		13.01 (12.2)
Median income		67,599.83 (18,390.1)
Level of family poverty		9.44 (4.2)

**Table 2:** Crude and adjusted association between food access and preterm birth.

Exposure Variable	Total Crude OR (95% C.I.)	Total Adjusted OR (95% C.I.)
	*n* = 7,533,319	*n* = 7,533,319
Food desert indicator (1 and 10)		
Low %	Ref	Ref
Mid-range %	1.08 (1.04–1.12)	1.04 (1.01–1.07)
High %	1.17 (1.14–1.22)	1.07 (1.03–1.11)

Note. Food desert indicator 1 and 10 refers to a low-income area with low access where the populace is at least 500 people and/or at least one-third of the census tract live >1 mile in urban communities or >10 miles in rural communities from a grocery store. The adjusted model includes maternal age, education level, prenatal care, previous preterm birth, urbanicity, family poverty percentage, and median income.

**Table 3:** Crude and adjusted association between food access and preterm birth by race.

	NH White *n* = 3,773,576		NH Black *n* = 1,050,925	
Exposure Variable	Crude	Adjusted	Crude	Adjusted
	OR (95% CI)	OR (95% CI)	OR (95% CI)	OR (95% CI)
Food desert indicator (1 and 10)				
Low %	Ref	Ref	Ref	Ref
Mid-range %	1.07 (1.04–1.11)	* 1.03 (1.00–1.04)	1.06 (1.00–1.12)	1.06 (1.01–1.11)
High %	1.15 (1.12–1.19)	1.06 (1.01–1.07)	1.16 (1.10–1.21)	1.10 (1.04–1.16)

Note. NH = non-Hispanic. Food desert indicator 1 and 10 refers to a low-income area with low access where the populace is at least 500 people and/or at least one-third of the census tract live >1 mile in urban communities or >10 miles in rural communities from a grocery store. The adjusted models include maternal age, education level, prenatal care, previous preterm birth, urbanicity, family poverty percentage, and median income. * *p* > 0.05.

## Data Availability

Birth records are available via an application to the NCHS. Food desert data are publicly available from the U.S. Department of Agriculture (USDA) Food Access Research Atlas.
